# Supplementary material for: Validity and internal consistency of EQ-5D-3L quality of life tool among pre-dialysis patients with chronic kidney disease in Sri Lanka, a lower middle-income country
Source: PLoS One. 2019 Jun 26;14(6):e0211604. doi: 10.1371/journal.pone.0211604 (PMC6594575; doi:10.1371/journal.pone.0211604)
Supplement: S2 Appendix — (DOCX) [file pone.0211604.s002.docx]

**S2 appendix : Subgroup analysis according to age**

**Age <59 versus Age >60**

**Table A: Correlation of SF-36 summary scores with EQ-5D-3L scores**

|  | **EQ-5D-3L index score**  **Spearman rho (p)** | | **EQ-5D-3L VAS score**  **Spearman rho (p)** | |
| --- | --- | --- | --- | --- |
|  | **Age <59** | **Age >60** | **Age <59** | **Age >60** |
| SF-36 Physical summary score | r_s_= 0.277  P< 0.001 | r_s_= 0.272  P< 0.001 | r_s_= 0.194  P< 0.001 | r_s_= 0.209  P< 0.001 |
| SF-36 Mental summary score | r_s_= 0.332  P< 0.001 | r_s_= 0.345  P< 0.001 | r_s_= 0.167  P< 0.001 | r_s_= 0.180  P< 0.001 |

**­­­­­­**

**Table B: Known group comparison among participant with or without depression with SF-36 summary scores and EQ-5D-3L scores**

|  | **Age <59** | | | **Age >60** | | |
| --- | --- | --- | --- | --- | --- | --- |
|  | With depression | Without depression | P value | With depression | Without depression | P value |
| EQ-5D-3L index score | 0.46  (0.34 to 0.68) | 0.75  (0.67 to 0.81) | <0.001 | 0.34  (0.31 to 0.58) | 0.75  (0.56 to 0.81) | <0.001 |
| EQ-5D-3L VAS score | 50.00  (40.00 to 60.00) | 60.00  (40.00 to 70.00) | <0.001 | 40.00  (40.00 to 50.00) | 60.00  (40.00 to 70.00) | <0.001 |
| SF-36 physical | 35.00  (24.38 to 41.25) | 40.58  (37.00 to 48.58) | <0.001 | 33.75  (25.00 to 40.00) | 36.25  (27.5 to 42.97) | 0.04 |
| SF-36 Mental | 38.23  (32.58 to 42.52) | 37.50  (31.87 to 53.12) | <0.001 | 38.00  (31.25 to 41.13) | 40.44  (37.22 to 44.35) | <0.001 |

**Table C: Known group comparison among participant with or without psychological distress with SF-36 summary scores and Q-5D-3L scores**

|  | **Age <59** | | | **Age >60** | | |
| --- | --- | --- | --- | --- | --- | --- |
|  | With distress | Without distress | P value | With distress | Without distress | P value |
| EQ-5D-3L index score | 0.55  (0.34 to 0.75) | 0.75  (0.63 to 1.00) | <0.001 | 0.35  (0.34 to 0.68) | 0.72  (0.41 to 0.79) | <0.001 |
| EQ-5D-3L VAS score | 50.00  (40.00 to 60.00) | 60.00  (40.00 to 80.00) | <0.001 | 40.00  (40.00 to 60.00) | 50.00  (40.00 to 70.00) | <0.001 |
| SF-36 physical | 34.37  (25.00 to 40.00) | 43.12  (33.75 to 58.75) | <0.001 | 33.75  (23.75 to 39.37) | 38.75  30.31 to 55.62) | <0.001 |
| SF-36 Mental | 38.48  (33.08 to 42.00) | 41.88  (38.12 to 57.00) | <0.001 | 38.25  (32.33 to 41.12) | 41.12  (36.85 to 48.95) | <0.001 |
