# Supplementary material for: Validity and internal consistency of EQ-5D-3L quality of life tool among pre-dialysis patients with chronic kidney disease in Sri Lanka, a lower middle-income country
Source: PLoS One. 2019 Jun 26;14(6):e0211604. doi: 10.1371/journal.pone.0211604 (PMC6594575; doi:10.1371/journal.pone.0211604)
Supplement: S3 Appendix — (DOCX) [file pone.0211604.s003.docx]

**S3 appendix : Subgroup analysis according to education level**

Education level up to Grade 5 versus Education level above Grade 5

**Table A: Correlation of SF-36 summary scores with EQ-5D-3L scores**

|  | **EQ-5D-3L index score**  **Spearman rho (p)** | | **EQ-5D-3L VAS score**  **Spearman rho (p)** | |
| --- | --- | --- | --- | --- |
|  | **Up to Grade 5** | **Above Grade 5** | **Up to Grade 5** | **Above Grade 5** |
| SF-36 Physical summary score | r_s_= 0.344  P< 0.001 | r_s_= 0.218  P< 0.001 | r_s_= 0.228  P< 0.001 | r_s_= 0.193  P< 0.001 |
| SF-36 Mental summary score | r_s_= 0.325  P< 0.001 | r_s_= 0.346  P< 0.001 | r_s_= 0.180  P< 0.001 | r_s_= 0.178  P< 0.001 |

**­­­­­­**

**Table B: Known group comparison among participant with or without depression with SF-36 summary scores and EQ-5D-3L scores**

|  | **Up to Grade 5** | | | **Above Grade 5** | | |
| --- | --- | --- | --- | --- | --- | --- |
|  | With depression | Without depression | P value | With depression | Without depression | P value |
| EQ-5D-3L index score | 0.34  (0.34 to 0.63) | 0.75  (0.58 to 1.00) | <0.001 | 0.38  (0.34 to 0.68) | 0.75  (0.66 to 0.81) | <0.001 |
| EQ-5D-3L VAS score | 40.00  (40.00 to 50.00) | 60.00  (40.00 to 70.00) | <0.001 | 40.00  (40.00 to 50.00) | 60.00  (40.00 to 77.50) | <0.001 |
| SF-36 physical | 33.75  (23.75 to 40.00) | 36.88  (30.00 to 47.50) | <0.001 | 35.00  (25.00 to 40.62) | 36.25  (30.00 to 49.84) | 0.04 |
| SF-36 Mental | 37.46  (30.41 to 41.33) | 39.87  (37.00 to 44.54) | <0.001 | 38.58  (33.42 to 41.82) | 41.00  (37.15 to 47.16) | <0.001 |

**Table C: Known group comparison among participant with or without psychological distress with SF-36 summary scores and EQ-5D-3L scores**

|  | **Up to Grade 5** | | | **Above Grade 5** | | |
| --- | --- | --- | --- | --- | --- | --- |
|  | With distress | Without distress | P value | With distress | Without distress | P value |
| EQ-5D-3L index score | 0.46  (0.34 to 0.75) | 0.73  (0.46 to 1.00) | <0.001 | 0.46  (0.34 to 0.75) | 0.75  (0.66 to 0.81) | <0.001 |
| EQ-5D-3L VAS score | 50.00  (40.00 to 60.00) | 50.00  (40.00 to 70.00) | <0.001 | 50.00  (40.00 to 60.00) | 60.00  (40.00 to 70.00) | <0.001 |
| SF-36 physical | 33.12  (23.59 to 38.75) | 41.25  (30.94 to 56.25) | <0.001 | 35.00  (25.00 to 40.00) | 42.50  33.75 to 58.75) | <0.001 |
| SF-36 Mental | 37.92  (31.15 to 41.12) | 41.12  (36.75 to 50.87) | <0.001 | 38.98  (34.46 to 41.94) | 41.33  (38.00 to 56.67) | <0.001 |
